# Pancreaticogastrostomy After Pancreatoduodenectomy

**DOI:** 10.1155/1992/96487

**Published:** 1992

**Authors:** A. Sauvanet, J. Belghiti, Y. Panis, B. Gayet, E. Camara, G. Urrejola, F. Fékété

**Affiliations:** Service de Chirurgie Digestive Université Paris VII Hôpital Beaujon 100 Boulevard du Général Leclerc Clichy 92110 France

## Abstract

The aim of this study was to evaluate the place of pancreaticogastrostomy (PG) in reducing pancreatic fistula after pancreatoduodenectomy. From January 1988 to June 1991, 32 consecutive patients (mean age, 57 years) were operated on, 25 for malignant disease (78%). The pancreatic remnant was normal in 17 patients (53%) and sclerotic in the others. There was one operative death (3.1%) unrelated to PG. Post-operative complications occurred in five patients (16%). Only two complications were related to PG: 1 patient had anastomotic intra-gastric bleeding and was reoperated on, 1 patient with a normal pancreatic remnant developed a pancreatic fistula (3.1%) treated conservatively.

Reported series of PG, as well as our results, demonstrates that PG is associated with a dramatic decrease of both pancreatic fistula and mortality rates. The risk of anastomotic haemorrhage can be reduced by preventative ligation of submucosal gastric vessels.

In conclusion, PG appears as a simple and reliable method of management of the pancreatic remnant after pancreatoduodenectomy.


*HPB Surgery*, 1992, Vol. 6, pp. 91–98
Reprints available directly from the publisher
Photocopying permitted by license only

© Harwood Academic Publishers GmbH
Printed in the United States of America

### PANCREATICOGASTROSTOMY AFTER PANCREATODUODENECTOMY

A. SAUVANET, J. BELGHITI, Y. PANIS, B. GAYET, E. CAMARA, G. URREJOLA and F. FÉKÉTÉ

*Service de Chirurgie Digestive, Université Paris VII, Hôpital Beaujon, 100 Boulevard du Général Leclerc, 92110 Clichy, France*

*(Received 12 November 1991)*

The aim of this study was to evaluate the place of pancreaticogastrostomy (PG) in reducing pancreatic fistula after pancreatoduodenectomy. From January 1988 to June 1991, 32 consecutive patients (mean age, 57 years) were operated on, 25 for malignant disease (78%). The pancreatic remnant was normal in 17 patients (53%) and sclerotic in the others. There was one operative death (3.1%) unrelated to PG. Post-operative complications occurred in five patients (16%). Only two complications were related to PG: 1 patient had anastomotic intra-gastric bleeding and was reoperated on, 1 patient with a normal pancreatic remnant developed a pancreatic fistula (3.1%) treated conservatively.

Reported series of PG, as well as our results, demonstrates that PG is associated with a dramatic decrease of both pancreatic fistula and mortality rates. The risk of anastomotic haemorrhage can be reduced by preventative ligation of submucosal gastric vessels.

In conclusion, PG appears as a simple and reliable method of management of the pancreatic remnant after pancreatoduodenectomy.

KEY WORDS: Pancreatoduodenectomy, pancreaticogastrostomy, pancreatic fistula

Pancreaticoduodenal resection is performed for treatment of both benign and malignant diseases of the periampullary region and head of the pancreas. One of the main problems after this resection concerns the management of the residual pancreatic stump. Pancreaticojejunostomy is the most often used procedure but its dehiscence occurs in about 10% of cases and can be associated with a specific morbidity and mortality[1–7]. Pancreaticogastrostomy (PG) was emphasized in recent series of pancreaticoduodenal resections reported without pancreatic fistula[8–11].

We report here our experience of this technique after pancreatoduodenectomy.

### PATIENTS AND METHODS

### *Patients*

From January 1988 to June 1991, 32 consecutive patients (22 males and 10 females) aged 35–69 years (average: 57 years) underwent a pancreatoduodenectomy. This resection was performed for the following diseases: adenocarcinoma of the pan-

Address correspondence to: Pr J. Belghiti, Hôpital Beaujon, Service de Chirurgie Digestive, 100 Boulevard du <Général Leclerc, 92110 Clichy, France creatic head ($n = 21$), carcinoma of the ampulla of Vater ($n = 3$), cystic dystrophy of aberrant pancreatic tissue ($n = 2$), chronic pancreatitis ($n = 4$), solitary metastasis of a renal adenocarcinoma ($n = 1$), injury of the duodenum with large defect during resection of a retroperitoneal sarcoma ($n = 1$). Pathological examination of the pancreatic parenchyma showed normal pancreatic tissue in 17 cases (53%) and sclerotic tissue in 15 (47%).

### *Operative Procedure*

The neck of the pancreas was transected to the left side of the portal vein. The pancreatic duct was identified and individual bleeeding vessels were ligated using single 6–0 polypropylene suture (Prolene®). The stump of the remaining pancreas was freed from retroperitoneal attachment for 2 cm. The posterior wall of the stomach was approximated to the pancreatic stump without tension and the site of the gastric incision was easily found on the antrum if a pylorus-preserving procedure was performed, as in 4 patients operated for benign diseases. After distal gastrectomy ($n = 28$), gastric mobilisation was often necessary in order to perform the PG on the fundus. The anastomosis was performed with 4–0 Prolene® continuous suture between the gastric seromuscular layer (after preventative ligation of submucosal gastric vessels) and the pancreatic parenchyma. No tube was left through anastomosis but care was taken to avoid pancreatic duct obliteration. Biliary and digestive tracts were restored by hepaticojejunal and gastrojejunal anastomosis to the first jejunal loop. Open drainage was always left in place near PG and choledocojejunostomy up to the 6th post-operative day; amylase level in the drainage fluid was assayed daily. A nasogastric tube was left in place until the return of intestinal activity. A postoperative oral radiologic study was performed only if a PG fistula was suspected on the clinical or biological data (amylase level in the drainage fluid more than 3 times normal serum amylase level).

### RESULTS

The postoperative course was uncomplicated in 26 patients (81%). Six patients had postoperative complications with one (3.1%) postoperative death.

One peritoneal haemorrhage due to slippage of the gastroduodenal artery ligature was the cause of the postoperative death (day 2). In this patient, no pancreatic complication was observed at emergency reoperation.

One patient developed postoperative pancreatitis diagnosed by elevation of serum amylase level and enlargement of the pancreatic stump on CT scan; this patient was successfully treated conservatively during 12 days by parenteral nutrition and somatostatin. Two patients developed sub-phrenic collections: one patient with infected biliary collection (*E. Coli*) was reoperated on at day 6; the other patient with a sterile serous collection was treated by percutaneous drainage on day 10. Amylase levels in these two collections was less than 3 times normal serum amylase level. One patient developed intra-luminal haemorrhage on the first postoperative day. Reoperation was performed and bleeding was located on the gastric side of the PG; hemostasis was obtained by vessel ligation through an anterior gastrotomy. This patient who was the first of our series didn't undergo preventative ligature of submucosal gastric vessels. In the 3 reoperated patients, the PG was healed.

Only one patient developed a pancreatic fistula on the 5th post-operative day; this fistula, drained by the operatively placed drains, closed after total parenteral nutrition and somatostatin until the 18th postoperative day. Thus, the overall pancreatic fistula rate of our series was 3.1%.

### DISCUSSION

After pancreatoduodenectomy with pancreaticojejunostomy, pancreatic fistula is the most serious postoperative complication observed. The rate of this complication is still close to 10%; when it occurs, it is often associated with a significant morbidity and an increased mortality rate[3–5,7,12–16]. In order to reduce the incidence and the severity of these fistulae, several techniques of pancreaticojejunostomy have been described including: (a) pancreatic, biliary and gastric anastomosis using the same first jejunal loop (Child's procedure) and its modification: end-to-end[1,12,16] or end-to-side anastomosis[14], with or without stent[3], or with a 60 cm interval between pancreatic and other anastomoses[6]; (b) anastomosis isolated on a separate Roux-en-Y loop[2]. Comparative analysis of these different procedures shows that pancreaticojejunostomy with 60 cm interval[6] or with isolated Roux-en-Y[2] seems to be associated with a lower mortality rate.

On the other hand, some authors have proposed to close the stump of the pancreatic remnant, sometimes after adhesive injection in the ductal system[2,17,18,19]. Comparison between pancreatic stump closure and pancreaticojejunostomy shows no difference in pancreatic fistula rate but a lower mortality rate with stump closure due to the absence of septic contamination[17].

Pancreaticogastrostomy, first described by Waugh and Clagett in 1946[20], was emphasized by recent and important series of pancreaticoduodenal resections reported without pancreatic fistula[8–11]. According to these favorable results, we decided to perform systematically this procedure with direct suture between the pancreatic stump and the posterior gastric wall. Some authors perform the anastomosis by intussusception of the pancreatic end into the gastric lumen[11]. PG is performed easily in association with preservation of the pylorus. When a simultaneous distal gastrectomy is performed, the anastomosis required a gastric mobilization by opening the gastrocolic ligament; however, PG is impossible in the case of a previous two-thirds gastrectomy. As shown in Table 1, a very low fistula rate after this procedure is reported. Among 177 patients, only two fistulae (1.1%) — including one of our patients — were reported; none of them required reoperation. The risk of pancreatic fistula (3.4%) is dramatically reduced by PG, although it persists in cases of a non-sclerotic pancreatic stump (i.e. pancreatic carcinoma) as in the two reported cases[21].

Both fistula and mortality rates after PG compare faborably with those reported with other procedures concerning the pancreatic stump[2,6,13,17,18]. Furthermore, PG is easier and less time-consuming to perform than isolated pancreaticojejunostomy and pancreatic exocrine function is not suppressed as in the case of closure of the pancreatic stump. The low rate of pancreatic fistula observed after PG could be explained by several factors: (a) the pancreas lies in a natural opposition to the

**Table 1** Reported series of pancreaticogastrostomy after pancreatoduodenectomy

| *Authors (ref)* | *Patients* | *Pancreatic fistula* | *Anastomotic haemorrhage* | *Mortality* |
|---|---|---|---|---|
| Mackie 1975 (21) | 25 | 1 | — | 2 |
| Flautner 1985 (9) | 27 | — | 1 | — |
| Kapur 1986 (11) | 31 | — | 2 | 2 |
| Icard 1988 (10) | 17 | — | 1 | — |
| Delcore 1990 (8) | 45 | — | 1 | 1 |
| Present study 1991 | 32 | 1 | 1 | 1 |
| Total | 177 | 2(1,1%) | 6(3,4%) | 6(3,4%) |

posterior gastric wall providing an anastomosis without tension; (b) gastric acidity inhibits the activation of pancreatic enzymes; (c) nasogastric aspiration provides decompression of the PG while equivalent precaution with pancreaticojejunostomy needs a Völker's drain[7]; (d) the thickness of the stomach wall provides a safer anastomosis than with jejunum.

Anastomotic haemorrhage, as we observed in our first patient, seems to be a specific complication of this procedure observed in 3.4% of cases[8–11,21]. It occurred mainly from the gastric wall, either with direct suture or with intussusception[9–11]. We have not observed this complication since we started performing a preventive ligation of the sub-mucosal gastric vessels.

Long-term permeability of pancreaticojejunostomy is uncertain[19]. Conversely the long term permeability of PG and a normal pancreatic exocrine function were demonstrated in patients who had undergone pancreatoduodenectomy for benign or low-malignant diseases[8,22]. In patients with chronic pancreatitis who underwent a side-to-side wirsungogastrostomy, a better long term pain control than with pancreaticojejunostomy has been reported[23]. The transient inactivation of pancreatic enzymes on the anastomotic site probably explains the best permeability of the anastomosis between pancreas and stomach.

In conclusion, our results support that pancreaticogastrostomy is a simple and safe alternative to pancreaticojejunostomy after pancreatoduodenectomy.

### *References*

1. Crist, D.W., Sitzmann, J.V. and Cameron, J.L. (1987) Improved hospital morbidity, mortality and survival after the Whipple procedure. *Ann. Surg.*, **206**, 358–365
2. Funovics, J.M., Karner, J., Pratschner, T. and Fritsch, A. (1989) Current trends in the management of carcinoma of the pancreatic head. *Hepatogastroenterol.*, **36**, 450–455
3. Grace, P.A., Pitt, H.Y., Tompkins, R.K., Denbesten, L. and Longmire, W.P. (1986) Decreased morbidity and mortality after pancreatoduodenectomy. *Am. J. Surg.*, **151**, 141–149
4. Lerut, J.P., Gianello, P.R., Otte, J.B. and Kestens, P.J. (1984) Pancreaticoduodenal resection. *Ann. Surg.*, **199**, 432–437
5. Papachristou, D.N. and Fortner, J.G. (1981) Pancreatic fistula complicating pancreatectomy for malignant disease. *Br. J. Surg.*, **68**, 238–240
6. Parc, R. and Herbière, P. (1983) Protection de l'anastomose pancréatico-jéjunale après duodéno-pancréatectomie céphalique pour tumeur. *Presse Med.*, **12**, 99–101
7. Trede, M. and Schwall, G. (1988) The complications of pancreatectomy. *Ann. Surg.*, **207**, 39–47
8. Delcore, R., Thomas, J.H., Pierce, G.E. and Hermeck, A.S. (1990) Pancreato-gastrostomy, a safe drainage procedure after pancreato-duodenectomy. *Surgery*, **108**, 641–647
9. Flautner, L., Timany, L.T. and Szelseny, A. (1985) Pancreatogastrostomy: an ideal complement to pancreatic head resection with preservation of the pylorus in the treatment of chronic pancreatitis. *Am. J. Surg.*, **150**, 608–611
10. Icard, P. and Dubois, F. (1988) Pancreaticogastrostomy following pancreatoduodenectomy. *Ann. Surg.*, **207**, 253–256
11. Kapur, B.M. (1986) Pancreaticogastrostomy in pancreaticoduodenal resection for ampullary carcinoma: experience with thirty-one cases. *Surgery*, **100**, 489–493
12. Braasch, J.W., Rossi, R.L., Watkins, E., Deziel, D.J. and Winter, P.F. (1986) Pyloric and gastric preserving pancreatic resection. *Ann. Surg.*, **204**, 411–418
13. Chapuis, Y., Yandza, T., Bonnichon, Ph., Delaitre, B. and Grateau, F. (1987) L'exclusion de l'anastomose pancréatico-jéjunale réduit-elle la mortalité de la duodéno-pancréatectomie céphalique. *Chirurgie*, **113**, 262–269
14. Funovics, J.M., Zoch, G., Wenzl, E. and Schulz, F. (1987) Progress in reconstruction after resection of the head of the pancreas. *Surg. Gynecol. Obstet.*, **164**, 545–548
15. Sato, T., Saitoh, Y., Moto, N. and Matsuno, S. (1977) Follow-up studies of radical resection for pancreatoduodenal cancer. *Ann. Surg.*, **186**, 581–587
16. Trede, M., Schwall, G. and Saeger, H.D. (1990) Survival after pancreatoduodenectomy. *Ann. Surg.*, **211**, 447–458
17. Di Carlo, V.D., Chiesa, R., Pontiroli, A.E., Carlucci, M., Staudacher, C., Zerbi, A., Cristallo, M., Braga, M. and Pozza, G. (1989) Pancreatoduodenectomy with occlusion of the residual stump by Neoprene injection. *World J. Surg.*, **13**, 105–111
18. Gall, F.P., Gebhart, C., Meister, R., Zirngibl, H. and Schneider, M.U. (1989) Severe chronic cephalic pancreatitis: Use of partial duodeno-pancreatectomy with occlusion of the pancreatic duct in 289 patients. *World J. Surg.*, **13**, 809–817
19. Goldsmith, H.S., Ghosh, B.C. and Huvos, A.G. (1971) Ligation versus implantation of the pancreatic duct after pancreaticoduodenectomy. *S G O*, **133**, 87–92
20. Waugh, J.M. and Clagett, O.T. (1946) Resection of the duodenum and head of the pancreas for carcinoma — an analysis of thirty cases. *Surgery*, **20**, 224–232
21. Mackie, J.A., Rhoads, J.E. and Park, C.D. (1975) Pancreaticogastrostomy: a further evaluation. *Ann. Surg.*, **181**, 541–545
22. Christiansen, J., Olsen, J.H. and Worning, H. (1971) The pancreatic function following subtotal pancreatectomy for cancer. *Scand. J. Gastroenterol. Suppl.*, **6**, 189–193
23. Pain, J.A. and Knight, M.J. (1988) Pancreaticogastrostomy: the preferred operation for pain relief in chronic pancreatitis. *Br. J. Surg.*, **75**, 220–222

*(Accepted by S. Bengmark 30 March 1992)*

### INVITED COMMENTARY

Sauvanet and colleagues report excellent results of pancreatogastrostomy after pancreatoduodenectomy in 32 consecutive patients. Their series includes one pancreatic fistula (3.1%), one anastomotic hemorrhage (3.1%) and one postoperative death (3.1%). These results are similar to their review of six reports since 1975 totalling 177 patients. In this collective review the reported rates of pancreatic fistula, anastomotic hemorrhage, and mortality were 1.1%, 3.4%, and 3.4%, respectively. This pancreatic fistula rate is significantly lower than the 10% to 20% rates reported with other methods of handling the pancreatic remnant.

Multiple techniques have been devised to deal with the pancreatic body and tail after resection of the head, suggesting that none is ideal. Surgical options include (1) a "dunking" end-to-end pancreatojejunostomy, (2) a mucosa-to-mucosa end-to-side pancreatojejunostomy, (3) a separate Roux-Y jejunal loop to the pancreas, (4) a 60cm interval between the pancreatic and other anastomoses, (5) pancreatogastrostomy, (6) closure of the pancreatic remnant without ductal occlusion, (7) occlusion of the pancreatic duct with various agents combined with closure of the pancreatic stump, and (8) total pancreatectomy.

With options 1 through 5 which include a pancreatic anastomosis, debate also continues on the advisability of stenting the anastomosis and, when a stent is used, whether it should be totally indwelling or extend externally. With options 6 to 8 which avoid a pancreatic anastomosis, an additional problem is pancreatic exocrine insufficiency and the life-long need for pancreatic enzyme replacement. In addition, with total pancreatectomy daily insulin is required, and recent evidence suggests that hepatic steatosis and even liver failure may be additional long-term concerns. Moreover, total pancreatectomy has not proven to improve survival in patients with pancreatic cancer. As a result, most groups world-wide with considerable experience have abandoned options which avoid a pancreatic anastomosis. Thus, the problem of pancreatic fistula after pancreatoduodenectomy remains a real issue that has yet to be solved.

Older literature clearly suggested a correlation between the rate of pancreatic fistulas and operative mortality. However, multiple reports from around the world have now documented mortality rates below 3% even with pancreatic fistula rates that remain in the 10% to 20% range. Multiple factors may explain this change including (1) the use of closed suction drains with monitoring for amylase to diagnose a fistula earlier, (2) the liberal use of computerized tomography (CT) scans postoperatively to diagnose pancreatitis or a perianastomotic collection, (3) CT-guided aspiration and percutaneous drainage of loculated intra-abdominal fluid collections, (4) an appreciation of the importance of nutritional status of the patient preoperatively, (5) the postoperative use of total parenteral nutrition in patients who develop a fistula, and (6) the routine use of prophylactic antibiotics and agents to prevent stress ulceration. Moreover, the use of somatostatin perioperatively is another more recent change in patient management that may further reduce the risk of pancreatic fistula and related mortality.

While the relationship between a pancreatic fistula and mortality is less clear now than in the past, a postoperative pancreatic fistula clearly increases hospital stay and cost. Thus, the multiple reports suggesting that pancreato-gastrostomy is associated with a very low pancreatic fistula rate must be examined more closely. In the report by Sauvanet and associates the authors monitored drain amylase and documented that intra-abdominal fluid collections requiring drainage were low in amylase. However, in most of the other reports claiming a low rate of pancreatic fistulas after pancreatogastrostomy, this information is not provided.

An additional theoretical concern with a pancreatogastrostomy that does leak is the added morbidity of a combined gastric and pancreatic fistula. However, since only two anastomotic leaks have been reported, this potential problem remains more theoretical than real. Anastomotic hemorrhage has also been reported in 3.4% of patients undergoing pancreatogastrostomy. While the incidence of this problem following pancreatojejunostomy is not well documented in the literature, it is probably less than 3%. In addition, when bleeding does occur after a pancreatojejunostomy, it usually comes from the pancreas rather than the jejunum. In comparison, the bleeds that have been reported after pancreatogastrostomy have mostly arisen from the gastric wall. Additional theoretical problems after pancreatogastrostomy that have not been adequately addressed in the available literature are delayed or precipitous gastric emptying and marginal ulceration.

Sauvanet *et al.* claim that pancreatogastrostomy is easier to perform than a separate Roux-Y jejunal limb to the pancreas. While this statement is probably true, most experienced pancreatic surgeons anastomose the pancreas, bile duct, and duodenum or stomach to the proximal jejunum without making a separate Roux-Y loop. Thus, in terms of operative time the pancreatogastrostomy provides no obvious advantage over the conventional pancreatojejunostomy. Moreover, a theoretical problem with a separate Roux-Y jejunal limb to the pancreas is an increased incidence of marginal ulceration. With the more conventional reconstruction, the presence of alkaline pancreatic secretions in that portion of jejunum anastomosed to the duodenum or stomach neutralizes gastric acid and lowers the incidence of marginal ulceration.

The physiologic effects of draining alkaline secretions directly into the stomach also need to be studied. How does pancreatogastrostomy affect the release of gastrin and secretin as well as other gastrointestinal hormones? Similarly, how does anastomosing the pancreas to the stomach affect the output of gastric acid and pancreatic juice? Moreover, are the answers to these questions influenced by preservation of the pylorus versus antrectomy with or without vagotomy? Most of these questions can be answered in animal studies that have yet to be, but should be, performed.

Despite these many unanswered questions regarding pancreatogastrostomy, present data suggest that this anastomosis is a legitimate rival to the more traditional pancreatojejunostomy. However, to convince many pancreatic surgeons that pancreatogastromy is the procedure of choice, careful physiologic studies in patients who have undergone this procedure should be undertaken. Moreover, to document that pancreatogastrostomy does have a lower pancreatic fistula rate, which should lead to reduced hospital stay and cost, a prospective, randomized trial versus pancreatojejunostomy needs to be performed.

H.A. Pitt
Department of Surgery
Johns Hopkins Medical Institutions
Baltimore, Maryland, USA

### INVITED COMMENTARY

In 1948 Waugh and Clagett described a technique of pancreaticogastrostomy for alimentary reconstruction after partial pancreatico-duodenectomy. The authors report to have used this technique with outstanding success in 32 patients. It should, however, be recalled that, according to the pathologist's report, the cut edges consisted of normal pancreatic tissue in only 17 cases, while sclerotic tissue had already developed in the remaining 15. Technically, this in itself reduces the risk of fistulation or pancreatitis by almost 50%.

The results reported by the authors appear to be excellent at first glance and, no doubt, reflect a high standard of technical excellence. Still they are best fair to middling in statistical terms, because latest mortality rates vary between 0.7% in the commentator's material and 0% in 118 consecutive pancreaticoduodenectomies done by Trede.

What, then, are the conclusions that can be drawn from the report?

Procedures for alimentary reconstruction after partial pancreatico-duodenectomy basically fall into one of three groups:

a. Procedures in which the pancreatic remnant is included in the passage of food (anastomosis to the stomach and Roux-en-Y anastomosis to the jejunum). This is the group to which the authors' technique belongs.

b. Procedures in which the pancreatic remnant is excluded from the passage of food (double Roux-en-Y anastomosis), but contributes its excretory function.

c. Complete exclusion of the pancreatic remnant in terms of ligation or obliteration of the pancreatic duct with or without fibrin sealant and with or without Ethi-block installation.

The most serious complications are partial pancreatitis involving the pancreatic remnant and, secondary to it, the development of a pancreatic fistula. But these are only relevant, if the ingested food passes through the critical segment with resultant isoperistaltic or antiperistaltic leakage ("fed fistula"). Inspite of the temptations of artistic surgery, preference should therefore be given to that technique which offers the greatest pathophysiologic benefits even if (surgically uncontrollable) fistulation does occur. Of the above procedures, the only candidates fulfilling these criteria are those listed under b. and c. With a view to preventing fistulation, total pancreatectomy, while still done occasionally, should be ranked among the obsolete procedures anyway.

In light of the above and in view of the comparatively small number of patients, the reported results are hardly likely to prompt any modification of the pathophysiologic concepts underlying pancreatic duct reconstruction. However, they document that, given a high standard of surgical skills, there still may be a place for the procedure at least until proven otherwise by controlled randomized trials. Considering that the outcome of surgery is generally satisfactory so that a large patient material would be needed for any such trial, this proof will not be furnished forthwith.

J. Funovics
Vienna

